# Loss of cardiomyocyte-specific adhesion G-protein-coupled receptor G1 (ADGRG1/GPR56) promotes pressure overload-induced heart failure

**DOI:** 10.1042/BSR20240826

**Published:** 2024-09-23

**Authors:** Jeanette Einspahr, Heli Xu, Rajika Roy, Nikki Dietz, Jacob Melchior, Jhansi Raja, Rhonda Carter, Xianhua Piao, Douglas G. Tilley

**Affiliations:** 1 Department of Cardiovascular Sciences, Aging + Cardiovascular Discovery Center, Lewis Katz School of Medicine, Philadelphia, PA, U.S.A.; 2 Department of Surgery, Duke University Medical Center, Durham, NC, U.S.A.; 3 Weill Institute for Neuroscience, University of California at San Francisco, San Francisco, CA, U.S.A.

**Keywords:** ADGRG1, Adhesion GPCR, cardiomyocytes, GPR56, heart failure, pressure overload

## Abstract

Adhesion G-protein-coupled receptors (AGPCRs), containing large N-terminal ligand-binding domains for environmental mechano-sensing, have been increasingly recognized to play important roles in numerous physiologic and pathologic processes. However, their impact on the heart, which undergoes dynamic mechanical alterations in healthy and failing states, remains understudied. ADGRG1 (formerly known as GPR56) is widely expressed, including in skeletal muscle where it was previously shown to mediate mechanical overload-induced muscle hypertrophy; thus, we hypothesized that it could impact the development of cardiac dysfunction and remodeling in response to pressure overload. In this study, we generated a cardiomyocyte (CM)-specific ADGRG1 knockout mouse model, which, although not initially displaying features of cardiac dysfunction, does develop increased systolic and diastolic LV volumes and internal diameters over time. Notably, when challenged with chronic pressure overload, CM-specific ADGRG1 deletion accelerates cardiac dysfunction, concurrent with blunted CM hypertrophy, enhanced cardiac inflammation and increased mortality, suggesting that ADGRG1 plays an important role in the early adaptation to chronic cardiac stress. Altogether, the present study provides an important proof-of-concept that targeting CM-expressed AGPCRs may offer a new avenue for regulating the development of heart failure.

## Introduction

Heart failure (HF) is a progressive disease expected to affect more than 8 million people in the United States by 2030 with care of these patients projected at approximately $70 billion annually [1]. Gold standard therapeutics for HF with reduced ejection fraction (HFrEF) consist of a small repertoire of neurohormone modulators, including antagonists of G-protein-coupled receptors (GPCRs), which remain the largest family of protein targets for approved drugs due to their impact on numerous pathophysiologic processes and relative ease of access at the cell surface [2]. Although the heart expresses hundreds of GPCRs [3], a majority of these lack defined roles in mediating cardiac homeostasis or remodeling responses to chronic stress. Adhesion GPCRs (AGPCRs), comprise 33 members in humans, contain large N-terminal adhesion domains that bind extracellular matrix proteins to sense mechanical changes to the environment [4]. While global deletion of a handful of AGPCRs has been reported to impart cardiac abnormalities, particularly during development [5–9], their expression in the heart and cardiomyocytes (CM) specifically, as well as their impact on cardiac health normally or during HF remains understudied. ADGRG1 (formerly known as GPR56) is widely expressed, with profound effects on several organs and diseases, including during brain development and cancer [10–13], and although cardiac ADGRG1 transcript expression was reported ∼25 years ago [5], its impact on cardiac function or remodeling during HF has not been investigated.

Although AGPCR ligands can be expressed in a tissue-specific manner, whether there are cardiac-specific ligands for ADGRG1 is not known; however, the extracellular matrix proteins collagen type III (COL3A1) and transglutaminase 2 (TG2) have each been identified to act as ligands for ADGRG1 [14,15] and each are known to be expressed in the heart [16,17]. Previous work focusing on the role of ADGRG1 in skeletal muscle found that it promotes primary myotube hypertrophy in response to stimulation with COL3A1 *in vitro*, while expression of both ADGRG1 and COL3A1 increase in a surgical model of hindlimb plantaris overload that induces skeletal muscle hypertrophy *in vivo* and that global deletion of ADGRG1 attenuates the mechanical overload-induced muscle hypertrophy [18]. An additional study also reported that ADGRG1 expression becomes elevated during human skeletal myoblast differentiation and that ADGRG1 deletion reduces myoblast fusion *in vitro*, thereby decreasing myotube size [19]. While these studies suggest ADGRG1 plays a role in mediating muscle growth in response to hypertrophic stress, whether this holds true for cardiac muscle is not known. However, knockdown of ADGRG1 in neonatal rat ventricular myocytes (NRVM) was reported to reduce angiotensin II-induced hypertrophy *in vitro* [20], suggesting that ADGRG1 may be capable of regulating cardiomyocyte response to chronic stress *in vivo*.

With expanding recognition that AGPCRs participate in regulating tissue responses to stress, and with their potential roles in the heart being vastly understudied, we aimed to explore their impact on cardiac processes. Here, we sought to investigate the impact of CM-specific ADGRG1 on cardiac structure and function normally and in response to HF. Herein, via use of a pressure overload model of HF in a newly generated CM-specific ADGRG1 KO mouse model, we have investigated for the first time the impact of CM-specific ADGRG1 on HF development, revealing that its deletion accelerates cardiac dysfunction, concurrent with blunted CM hypertrophy and enhanced cardiac inflammation, suggesting that ADGRG1 may play an important role in the early adaptation to chronic cardiac stress.

## Methods

### Animals

Animal experiments were conducted in the Lewis Katz School of Medicine under the National Institutes of Health (NIH) Guide for the Care and Use of Laboratory Animals and was approved by the Institutional Animal Care and Use Committee (IACUC) at Temple University with Animal Protocols 4891 and 4902. Wild-type (C57BL6/J) mice were attained from The Jackson Laboratory. Mice harboring *loxP* sites (ADGRG1^f/f^ [21]) were crossed with B6.FVB-Tg(Myh6-cre)2182Mds/J mice containing α-myosin heavy chain (Myh6) promotor-driven expression of cre (αMHC-Cre+, Strain #011038, attained from The Jackson Laboratory) to produce mice with constitutive cardiomyocyte-specific deletion of *Adgrg1* (CM-ADGRG1-KO). Numbers of mice used are listed in figure legends, with male and female mice indicated. All mice were genotyped using flox site primers (P2:5′–GGTGACTTTGGTGTTCTGCACGAC–3′, P4:5′–CACGAGACTAGTGAGACGTGCTAC–3′, and P6:5′–TGGTAGCTAACCTACTCCAGGAGC–3′) and Cre-specific primers (1468:5′–GGCGTTTTCTGAGCATACCT–3′ and 1469:5′–CTACACCAGAGACGGAAATCCA–3′) prior to experimental procedures. Both male and female mice were enrolled in studies unless otherwise indicated in figure legends.

### Transverse aortic constriction surgery

Transverse aortic constriction (TAC) was performed on 12-week-old mice anesthetized via continuous 3% isoflurane inhalation in 100% oxygen induction chamber as previously described [22]. Anesthetized mice were transferred to a nose cone and body temperature was maintained using a heating pad and rectal thermometer. The chest cavity was entered via partial thoracotomy to the second rib. The aortic arch was exposed, and the transverse aorta was constricted between the brachiocephalic and left common carotid artery by tying a 6–0 suture against a 27-gauge needle. Sham mice serve as surgical controls by entry to the chest cavity and aorta exposure, but no suture was secured. Post-operative subcutaneous administration of buprenorphine was given for analgesia. At study endpoints, mice were killed via isoflurane overdose, sacrificed and hearts were removed for further analysis.

### Adult mouse cardiomyocyte isolation

Cardiomyocytes were isolated from adult mice left ventricle as previously described [23]. Briefly, hearts were excised, and a cannula was placed through the aorta and perfusion buffer diffused through the heart for 5 min (speed 3 ml/min), followed by 12-min enzymatic digestion with buffer containing Collagenase B (Sigma-Aldrich, Cat#11088831001). The digested heart and sutures were removed from the cannula, the aorta and atria removed, left ventricle tissue broken apart further using a pipet and the cell suspension incubated at 37°C for 5 min for further digestion. Digestion was completed with the addition of a stopping buffer containing 5% fetal bovine serum (FBS). Following digestion, cells are washed in PBS, pelleted and flash frozen.

### Echocardiography

Cardiac function was evaluated as previously described [24] using the VisualSonic Vevo 2100 Imaging System (FUJIFILM). Hair removal from the chest was performed prior to image acquisition using nair. All mice were initially anesthetized with 3% isoflurane inhalation and subsequently reduced to 1% isoflurane for heart rate maintenance (HR was maintained between 400 and 500 bpm). Mice were placed in the supine position on the stage and echocardiography gel placed on the chest. The MS550D probe was used for long and short axis image acquisition in both B and M mode. Images were analyzed using the cardiac package and LV trace function in VevoLab 5.7.1 software. Echocardiographic parameters were measured and calculated as previously reported [25] and heart failure with reduced ejection fraction (HFrEF) was defined as %EF<40% [26]. Doppler echocardiography was performed at 1-week post-TAC using a MS250 probe for image acquisition of the aorta region. Images were analyzed using the cardiac package and AoV Flow and AoV VTI trace in VevoLab 5.7.1 software.

### Immunofluorescence (IF) and immunohistochemistry (IHC) of cardiac sections

IF and IHC were performed as previously described [25]. Briefly, the mouse chest cavity was exposed, abdominal aorta cut, hearts perfused with 25 mM KCl, PBS and subsequently excised and fixed in 4% paraformaldehyde (PFA). Fixed hearts were processed using a tissue processer, embedded in paraffin, and sectioned at 5 μm thickness on to charged microscope slides (Globe Scientific, Cat#1358W). Slides containing three heart sections were deparaffinized in xylene (3 × 5 min) and ethanol (3 × 100%, 1 × 90%, 1 × 80% and 1 × 70% sequentially, 5 min) prior to staining. To assess cardiomyocyte cross-sectional area, hearts were stained overnight at 4°C with Wheat Germ Agglutinin (20 μg/ml, Vector Labs, Cat#FL-1021), rinsed with PBS, and co-stained with DAPI NucBlue Fixed Cell ReadyProbes (ThermoFisher, Cat# R37606) for 10 min at room temperature (RT). To assess α-smooth muscle actin (αSMA) relative flourescence, hearts were stained overnight at 4°C with anti-αSMA (1:1000, Sigma, Cat#A2547), rinsed with PBS, and stained with secondary antibody Alexa647 (1:500, Goat anti-mouse) and co-stained with DAPI NucBlue for 10 min at room temperature. Image acquisition for IF was performed using the EVOS M7000 Fluorescence Microscope System. Analysis was performed using ImageJ. Relative αSMA fluorescence was determined by taking the average αSMA fluorescence normalized by DAPI fluorescence, quantified from 8 to 10 HVF/images of the left ventricle. To assess CD45+ cells, hearts were stained overnight at 4°C with anti-CD45+ (1:250, R&D Systems, Cat#AF114), rinsed with PBS, and secondary antibody Alexa647 (1:250, Donkey anti-goat) and co-stained with DAPI NucBlue for 10 min at room temperature. All IF slides were mounted and preserved using Prolong Gold Antifade Mountant (ThermoFisher, Cat#P36934).

To assess fibrotic lesion formation, Masson’s trichrome IHC was performed. Slides were placed in Bouin’s solution (Sigma-Aldrich, Cat#HT10132-1L) overnight at room temperature. Slides were rinsed, incubated in Weigert’s Iron Hematoxylin (Sigma-Aldrich, Cat#HT1079-1SET), Biebrich Scarlet-acid Fucshin (Sigma-Aldrich, Cat#HT151-250ML), with washes in double deionized water as needed. Sections were then placed in phosphotungstic-phosphomolybdic acid (Sigma-Aldrich, Cat#HT152-250ML), counterstained with Aniline Blue (Sigma-Aldrich, Cat#HT154-250ML), placed in 1% acetic acid and dehydrated again in 95% and 100% ethanol and xylene. All IHC slides were mounted and preserved with Permount Mounting Media (Fisher Scientific, Cat#SP15-100). Image acquisition for IF was performed using the EVOS M7000 Fluorescence Microscope System or Nikon Eclipse Ti-E. Image acquisition for IHC was performed using the Nikon Eclipse Ni-E. Cross-sectional area analysis was performed using NIKON Software. CD45 and Masson’s Trichrome analysis was performed using ImageJ. Average number of CD45 cells per high visual field (HVF) was determined by taking the average number of double positive (CD45 and Dapi) over total Dapi cells, quantified from 8 to 10 HFV/images of the left ventricle.

### Reverse transcription-quantitative polymerase chain reaction (RT-qPCR)

Total RNA was isolated from LV tissue samples using the PureLink RNA mini kit (Invitrogen, Cat#12183025), according to the manufacturer’s instructions. RNA yield was assessed using a Nanodrop 2000 (Thermo Scientific), and 1000 ng of RNA was input for cDNA synthesis using the High-Capacity cDNA Reverse Transcription kit (ThermoScientific, Cat#4368813). RT-qPCR was performed with SYBR Select Master Mix in triplicate using the primers listed in Supplementary Table S1. Data were analyzed using the Comparative CT Method (2^−∆∆CT^) and represented as a fold change normalized to translationally controlled tumor protein 1 (TPT1).

### Protein preparation and immunoblotting

Samples were lysed in RIPA buffer (1 M Tris pH 7.4, 10% NP-40, 10% sodium deoxycholate, 1 M sodium chloride, 0.5 M EDTA pH 8, 0.5 M sodium fluoride) containing 1X HALT protease inhibitor cocktail (ThermoFisher) and phosphatase inhibitor cocktail set IV (Calbiochem). Normalized protein concentrations of 50 μg (as determined via BCA assay) and equal volumes of lysates were subjected to SDS-polyacrylamide gel electrophoresis (10 or 12% gels) and subsequently transferred onto Immobilon-PSQ polyvinylidene fluoride 0.2 µm pore membranes (Millipore, Burlington, MA, U.S.A.). Membranes were incubated in blocking buffer (Rockland, Pottstown, PA, U.S.A.), and then probed for antibodies of interest. Primary antibodies used include Anti-GPR56 N-terminal antibody (1:1000, mouse, Sigma, MABN310), and GAPDH (1:1000, rabbit, Cell Signaling, 14C10). After washing membranes with TBS-T, they were subjected to secondary antibody at room temperature for 1 h in the dark (1:15000, IRDye680 Donkey anti-rabbit IgG, or IRDye800 Goat anti-mouse IgG, LI-COR Biosciences). Membranes were then visualized on the LI-COR Biosciences Odyssey System. Immunoblot densitometry was performed using Image Studio Lite software to normalize ADGRG1 expression (75 kDa species) to GAPDH expression (36 kDa, housekeeping protein).

### Single-cell RNA-sequencing analysis

Publicly available raw single-cell RNA sequencing data (unique molecular identifier [UMI] counts) were downloaded from the Gene Expression Ominbus (mouse GSE120064), with metadata including each cell id, its corresponding sample information and its cell type based on the expression of known markers, as described by the data contributors [27]. Cardiomyocytes were extracted from the full dataset based on the metadata, normalized and integrated with Seurat [28] to minimize batch effects across different conditions/groups, followed by clustering of different cell types using Uniform Manifold Approximation and Projection (UMAP) dimensional reduction [29]. For visualization purposes, counts from each dataset were interpreted with the use of Shiny Apps and single-cell data scaled and graphically represented using R ggPlot2.

### Statistical analysis

Statistical analysis and graph generation was performed, unless otherwise stated, using GraphPad Prism 10 software (GraphPad Software Inc., San Diego, CA, U.S.A.). Refer to figure legends for specific details regarding graphs and individual sample numbers per group. Statistical comparisons were made using two-tailed unpaired *t*-tests for mean of two groups, ordinary one-way ANOVA with Tukey’s Post Hoc for mean of three or more groups, two-way ANOVA with Tukey’s Post Hoc to compare two independent variables on a dependent variable and Log-rank (Mantel-Cox) test for survival comparison. In the case that an ANOVA test with Tukey’s post hoc analysis did not indicate significance but the associated Bartlett’s test yielded a *P-*value < 0.05, suggesting unequal variance that could lead to a Type 2 error, we performed Brown–Forsythe and Welch’s ANOVA tests with Dunnett’s T3 multiple comparison tests. All differences were considered significant at *P*<0.05.

## Results

### Cardiac and cardiomyocyte-specific ADGRG1 expression becomes decreased in response to pressure overload

**Figure 1:**
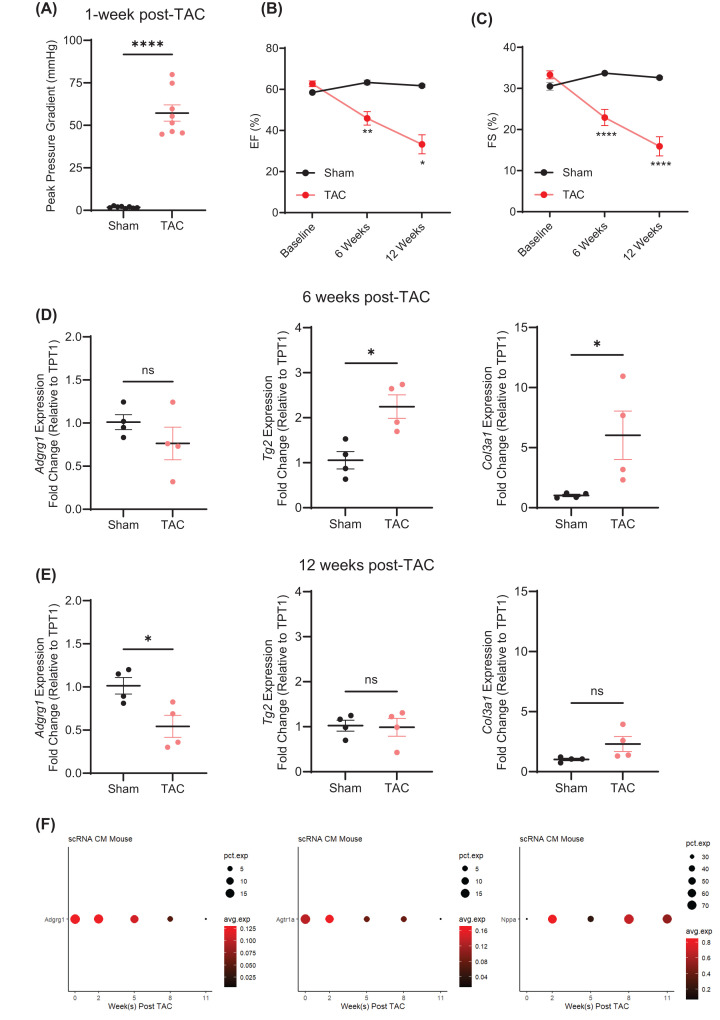
ADGRG1 and its ligands undergo dynamic alterations in expression in the heart during the development of heart failure (**A**) Doppler echocardiography was performed 1-week post-TAC surgery to assess peak pressure gradients at the constriction site in C57BL/6 mice. Data are mean ± SEM, ****P <0.0001, two-tailed *t*-test, *n* = 8 male mice per group. Serial echocardiography was performed at baseline, 6- and 12-week post-surgery to assess ejection fraction (%EF, **B**) and fractional shortening (%FS, **C**). Data are mean ± SEM, *P<0.05, **P<0.01, ****P<0.0001, two-way ANOVA with Tukey’s post-hoc test, *n*=8 (baseline, 6 weeks) or 4 (12 weeks) for Sham and TAC groups. Hearts were harvested at 6 weeks (**D**) or 12 weeks (**E**) post-surgery and LV processed for RNA extraction and RT-qPCR analysis of *Adgrg1*, *Tg2* and *Col3a1*. Data are mean ± SEM, *P<0.05, ns = not significant, two-tailed *t*-test, *n* = 4 male mice per group. (**F**) To investigate the expression of *Adgrg1* in cardiomyocytes specifically following TAC, we analyzed existing single cell RNA-sequencing data (GSE120064) from 2 to 4 male C57BL/6 male mice per timepoint [27]. Dot plots indicate the expression (in TP10K) and % cardiomyocyte expression of *Adgrg1* in response to TAC over time (compared with *Agtr1a*, and with *Nppa* as a control for TAC-induced changes in transcript expression).

To investigate the impact of the development of HF on cardiac ADGRG1 expression, we performed TAC surgery, a model of pressure overload-induced hypertrophy and subsequent development of HFrEF [25,30,31], on wild-type mice (C57BL6/J), as confirmed via pressure gradient analysis at 1-week post-TAC (Figure 1A) and via serial echocardiography, which showed decreases in % ejection fraction (%EF, Figure 1B) and % fractional shortening (%FS, Figure 1C), as well as increases in LV volumes and internal diameters (Supplementary Figure S1D–G). RT-qPCR analysis revealed that although *Adgrg1* expression was not altered by 6 weeks post-TAC, it’s known ligands transglutaminase 2 (*Tg2*) and type III collagen (*Col3a1*) were significantly elevated (Figure 1D). However, by 12-week post-TAC, *Adgrg1* expression was significantly reduced, while *Tg2* and *Col3a1* levels were comparable to sham controls (Figure 1E). Via analysis of previously published single cell-RNASeq data [27], we next assessed whether expression of *Adgrg1* specifically in cardiomyocytes becomes altered during the development of HF in response to TAC. Consistent with our LV expression data, *Adgrg1*, expressed at a level of 0.1286 TP10K in ∼20% of adult mouse cardiomyocytes at baseline, was reduced by 11-week post-TAC, to a level of 0.0046 TP10K in ∼2% of cardiomyocytes (Figure 1F, with *Nppa* expression shown as a known TAC-responsive transcript). This level of expression and response to TAC-induced cardiac stress is similar to that of the angiotensin II type 1a receptor (*Agtr1a*), which we have previously shown capable of inducing changes in cardiomyocyte function in response to stimulation [32]. Together, these data reveal that *Adgrg1* and its ligands are expressed in the heart, undergoing dynamic alterations in response to chronic stress, and *Adgrg1* expression in CMs becomes reduced upon development of HF.

### Cardiomyocyte-specific ADGRG1 deletion leads to changes in systolic and diastolic cardiac dimensions at baseline

**Figure 2:**
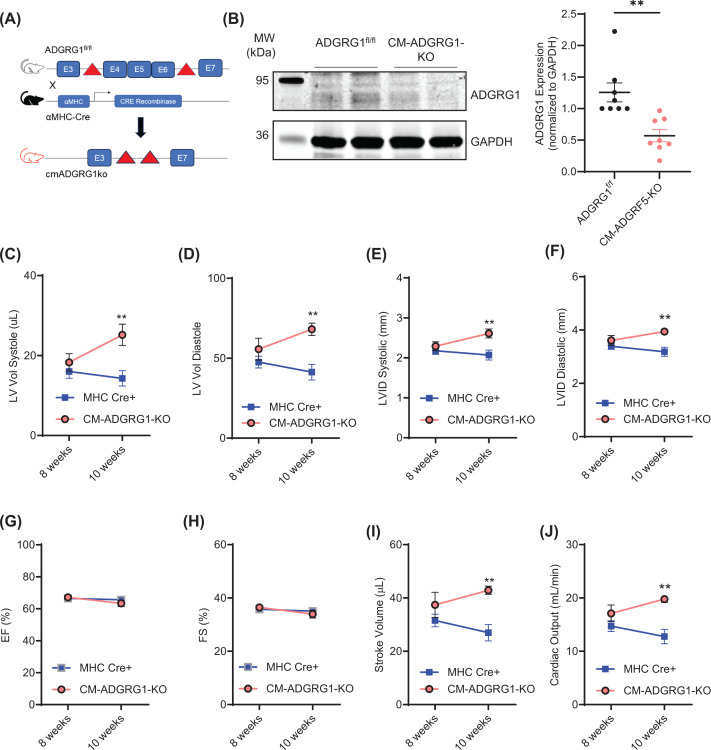
Constitutive cardiomyocyte-specific ADGRG1 deletion (**A**) ADGRG1 floxed mice (ADGRG1^fl/fl^), possessing a loxP site upstream of exon 4 as well as a 3′ loxP site downstream of exon 6, were crossed with transgenic mice expressing an αMHC promoter-driven Cre recombinase (αMHC-Cre) to generate mice with constitutive cardiomyocyte-specific deletion of ADGRG1/GPR56 (CM-ADGRG1-KO). (**B**) ADGRG1 knockdown was validated in isolated adult mouse cardiomyocytes (AMCM) by Western blot analysis. Data are mean ± SEM, **P<0.01, two-tailed *t*-test, *n* = 8 AMCM preparations (from 4 male and 4 female mice) per genotype. Echocardiography was used to assess systolic (**C**) and diastolic (**D**) LV volume, and systolic (**E**) and diastolic (**F**) internal diameter, ejection fraction (%EF, **G**), fractional shortening (%FS, **H**), stroke volume (**I**) and cardiac output (**J**) in 8- and 10-week-old CM-ADGRG1-KO (*n* = 5; 4 female, 1 male) and αMHC-Cre mice (*n* = 5; 3 female, 2 male). Data are mean ± SEM, **P<0.01, two-way ANOVA with Tukey’s post-hoc test.

Since ADGRG1 was previously shown to regulate aspects of skeletal myocytes [28,29], we sought to define the impact of CM-specific ADGRG1 on the heart. Thus, we crossed floxed ADGRG1 mice (ADGRG1^f/f^) [21] with αMHC-Cre mice, generating CM-specific ADGRG1 KO mice (CM-ADGRG1-KO) (Figure 2A,B). Subsequently, adult CM-ADGRG1-KO mice were characterized via echocardiography to determine whether loss of CM-specific *Adgrg1* impacted cardiac structure or function in the absence of stress. While CM-ADGRG1-KO mice did not display abnormalities in any of the echocardiographic parameters at 8 weeks of age versus αMHC-Cre controls, by 10 weeks of age they displayed small but significant increases in both systolic and diastolic LV volumes and internal diameters (Figure 2C–F); however, since both systolic and diastolic parameters increased, there were no differences in %EF or %FS (Figure 2G,H). With a larger increase in diastolic than systolic dimensions, both stroke volume and cardiac output were increased in CM-ADGRG1-KO mice at 10 weeks of age (Figure 2I,J). Notably, despite the alterations in systolic and diastolic cardiac volumes and internal diameters, CM-ADGRG1-KO mice did not display changes in LV wall thicknesses (Supplementary Figure S2B–E). Additionally, 12-week-old CM-ADGRG1-KO and αMHC-Cre mice were sacrificed for gravimetric analysis of their hearts, wherein no notable differences were observed in heart weight:tibia length ratios (Supplementary Figure S2G).

### Cardiomyocyte-specific ADGRG1 deletion accelerates cardiac dysfunction in response to pressure overload

**Figure 3:**
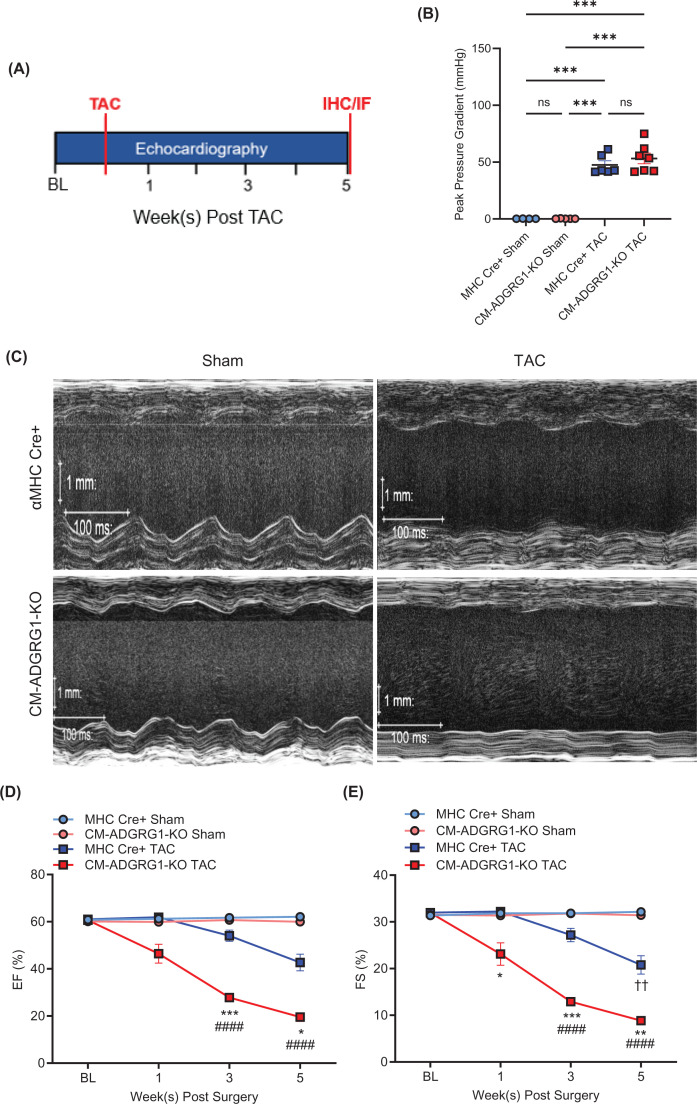
Cardiomyocyte-specific ADGRG1 deletion hastens cardiac dysfunction in response to chronic pressure overload (**A**) Schematic of TAC study design. Mice were 12 weeks of age at time of TAC surgery. (**B**) To confirm equivalent surgical constrictions *in vivo*, doppler echocardiography was performed 1-week post-surgery to assess peak pressure gradient of the constriction site. Data are mean ± SEM, ***P<0.001, ns = not significant; Bartlett’s test associated with initial one-way ANOVA yielded *P*<0.0001 suggesting unequal variance, thus Brown–Forsythe and Welch’s ANOVA tests with Dunnett’s T3 multiple comparison tests were performed. (**C**) Echocardiography was performed to assess % ejection fraction (**D**) and % fractional shortening (**E**). Data are mean ± SEM, *P<0.05, **P<0.01, ***P<0.001 versus MHC-Cre TAC, ####*P*<0.0001 versus CM-ADGRG1-KO Sham and ††*P*<0.01 versus MHC-Cre Sham, two-way ANOVA with Tukey’s post-hoc test. *n* = 4 (MHC-Cre Sham; 2 males, 2 females), *n* = 5 (CM-ADGRG1-KO Sham; 1 male, 4 females), *n* = 6 (MHC-Cre TAC; 3 males, 3 females), *n* = 7 (CM-ADGRG1-KO TAC; 2 males, 5 females).

We next subjected CM-ADGRG1-KO mice to TAC surgery to determine whether cardiomyocyte-specific loss of ADGRG1 impacts cardiac function or remodeling outcomes in response to chronic stress (Figure 3A,B). Via serial echocardiography, we observed that CM-ADGRG1-KO mice experienced an accelerated increase in LV dilation (Figure 3C), particularly driven by large increases in systolic LV volume and internal diameter (Supplementary Figure S3D,F) resulting in substantial reductions in both %EF (Figure 3D) and %FS (Figure 3E). These data indicate that CM-ADGRG1-KO mice experience an accelerated development of cardiac dysfunction in response to pressure overload-mediated chronic stress. Notably, while the αMHC-Cre control mice experienced a transient increase in LV wall thickness at the 3-week post-TAC timepoint, consistent with pro-hypertrophic compensation against pressure overload-induced stress at this timepoint [30,31], the CM-ADGRG1-KO mice lacked this response (Supplementary Figure S3H,I).

**Figure 4:**
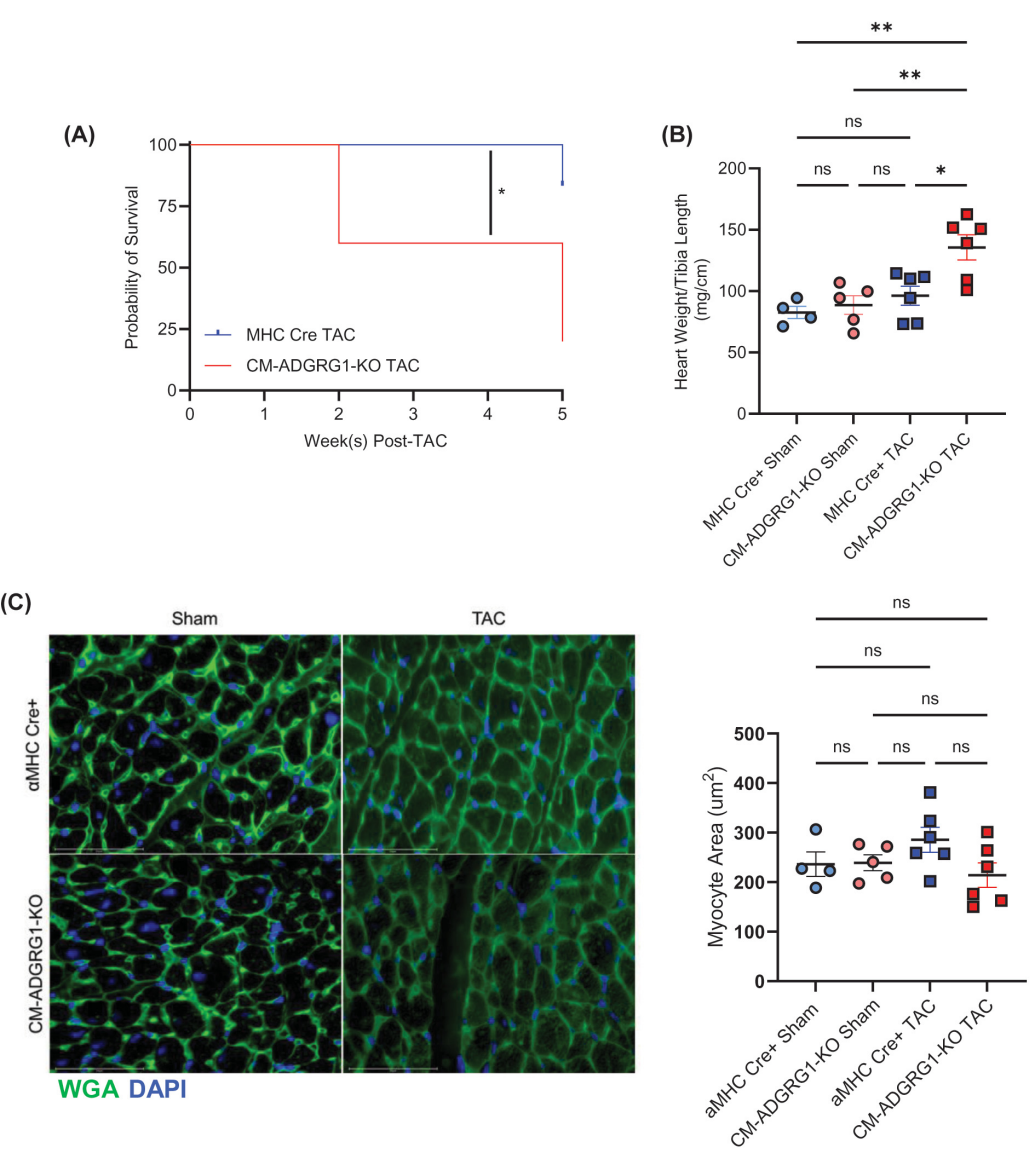
Cardiomyocyte-specific ADGRG1 deletion reduces survival in response to chronic pressure overload (**A**) Survival analysis of CM-ADGRG1-KO versus MHC Cre mice following TAC. *P<0.01, Log-rank (Mantel-Cox) test, *n* = 11 (CM-ADGRG1-KO; 6 males, 5 females) and 6 (MHC Cre; 4 males, 2 females). (**B**) Gravimetric data was collected from 5-week-post TAC mice and Heart Weight/Tibia Length ratios are indicated. Data are mean ± SEM, *P<0.05, **P<0.01, ns = not significant, one-way ANOVA with Tukey’s post-hoc test. *n* = 4 (MHC-Cre Sham; 2 males, 2 females), *n* = 5 (CM-ADGRG1-KO Sham; 1 males, 4 females), *n* = 6 (MHC-Cre TAC; 3 males, 3 females), *n* = 6 (CM-ADGRG1-KO TAC; 2 males, 4 females). (**C**) Wheat germ agglutinin (WGA) staining was performed on cardiac slices from CM-ADGRG1-KO and MHC Cre mice at 5-week post-surgery, with data quantified in histogram. Data are mean ± SEM, ns = not significant, one-way ANOVA with Tukey’s post-hoc test. *n* = 4 (MHC-Cre Sham; 2 males, 2 females), *n* = 5 (CM-ADGRG1-KO Sham; 1 male, 4 females), *n* = 6 (MHC-Cre TAC; 3 males, 3 females), *n* = 6 (CM-ADGRG1-KO TAC; 2 males, 4 females).

**Figure 5:**
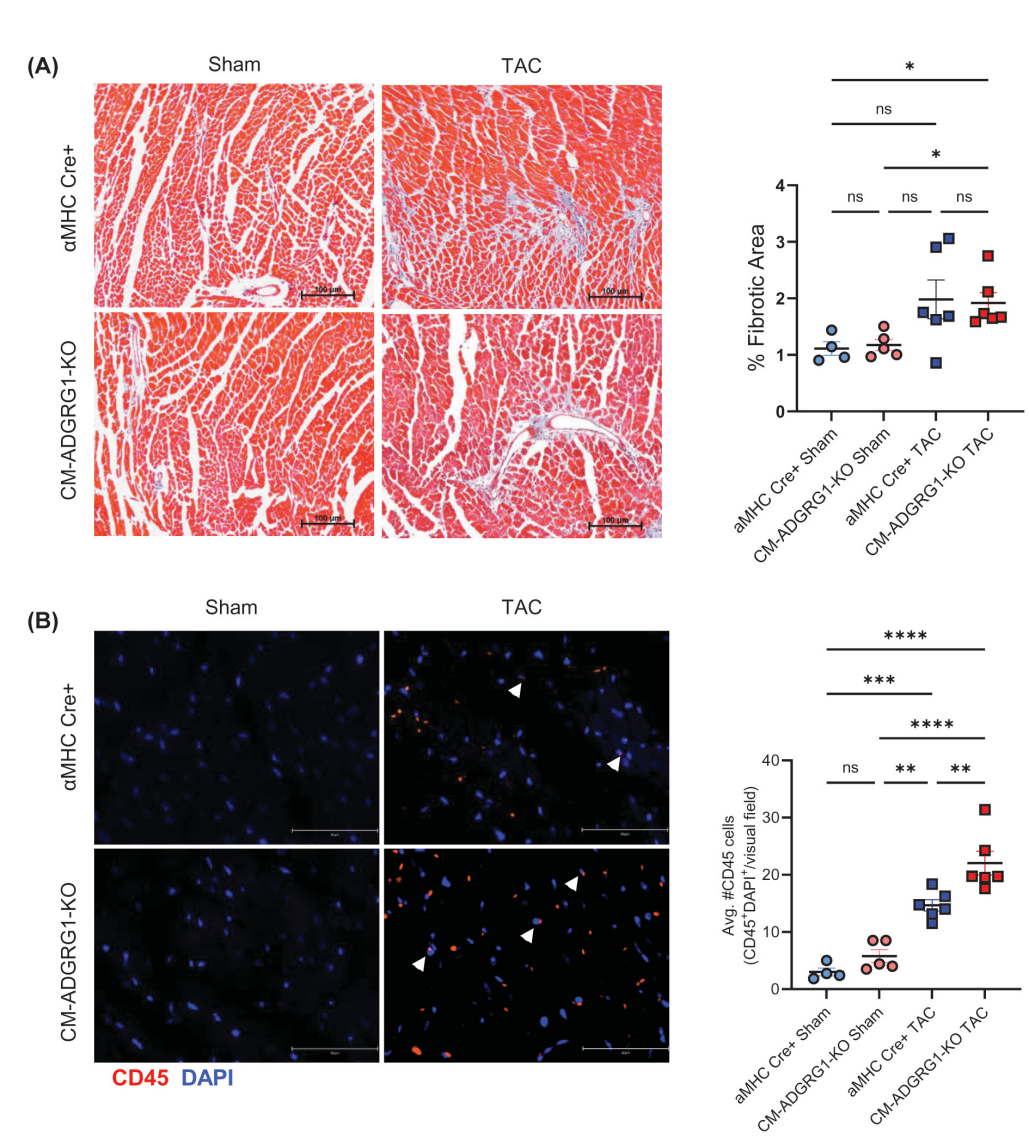
Cardiomyocyte-specific ADGRG1 deletion increases peripheral immune cells recruitment to the heart in response to chronic pressure overload (**A**) Masson’s trichrome (MTC) staining was performed on cardiac slices from CM-ADGRG1-KO and MHC Cre mice at 5-week post-surgery, with data quantified in histogram. Data are mean ± SEM, *P<0.05, ns = not significant; Bartlett’s test associated with initial one-way ANOVA yielded *P*=0.0381 suggesting unequal variance, thus Brown–Forsythe and Welch’s ANOVA tests with Dunnett’s T3 multiple comparison tests were performed. *n* = 4 (MHC-Cre Sham; 2 males, 2 females), *n* = 5 (CM-ADGRG1-KO Sham; 1 male, 4 females), *n* = 6 (MHC-Cre TAC; 3 males, 3 females), *n* = 6 (CM-ADGRG1-KO TAC; 2 males, 4 females). (**B**) CD45 (red) and DAPI (blue) staining were performed on cardiac slices from CM-ADGRG1-KO and MHC Cre mice at 5-week post-surgery, with data quantified in histogram. Data are mean ± SEM, **P<0.01, ***P<0.001, ****P<0.0001, ns = not significant, one-way ANOVA with Tukey’s post-hoc test. *n* = 4 (MHC-Cre Sham; 2 males, 2 females), *n*=5 (CM-ADGRG1-KO Sham; 1 male, 4 females), *n*=6 (MHC-Cre TAC; 3 males, 3 females), *n*=6 (CM-ADGRG1-KO TAC; 2 males, 4 females).

Coinciding with the worsened cardiac dimensions over time, CM-ADGRG1-KO mice experienced worse survival compared to the αMHC-Cre control group (Figure 4A), such that experiments were terminated by 5-week post-TAC to be able to perform gravimetric, IHC and IF analyses of the hearts. Gravimetric assessment of heart size at 5-week post-TAC showed significantly higher heart weights in the CM-ADGRG1-KO versus αMHC-Cre mice (Figure 4B), indicating that CM-ADGRG1-KO mice may experience an accelerated development of cardiac dysfunction. To determine whether maladaptive cardiac remodeling processes were augmented in the CM-ADGRG1-KO mice, we performed several immunohistochemical analyses. Notably, and despite increased heart weight/tibia lengths, wheat germ agglutinin staining revealed no significant differences in cardiomyocyte hypertrophy in either group at 5 weeks post-TAC, though there was a trend toward less cardiomyocyte cross-sectional area in the CM-ADGRG1-KO group (Figure 4C). LV fibrosis was significantly elevated by 5 weeks post-TAC in the CM-ADGRG1-KO group but was not different from the αMHC-Cre TAC group (Figure 5A), nor was there a difference in αSMA staining amongst any of the groups (Supplementary Figure S4). Finally, we stained the LV sections for CD45, a marker of peripheral immune cells. While CD45^+^ cells were increased in both TAC groups, there was a significantly higher infiltration of CD45^+^ cells at 5-week post-TAC in the CM-ADGRG1-KO mice (Figure 5B), indicating an augmented cardiac inflammatory response. Of note, we did not detect significant differences in any of the gravimetric, IHC or IF data between the CM-ADGRG1-KO and αMHC-Cre Sham controls, confirming that despite the changes in baseline LV volume and internal diameters (Supplementary Figure S3D–G), loss of ADGRG1 in the absence of stress does not induce overt pathological remodeling.

## Discussion

Recognition of AGPCRs as important mediators of mechanical stress signaling across various tissues and disease states has steadily increased over the last two decades, but their impact on cardiac structure and function normally and during HF remains vastly understudied compared with their roles in other organs and diseases [2,11]. ADGRG1 has long been identified to be a key regulator of cortical development and lamination, particularly in regulating oligodendrocyte precursor cell-mediated myelination [33,34], critical functions in cancer such as cell proliferation and migration or metastasis [10,15,35] and also has been shown to respond to shear stress in platelets to regulate hemostasis [36]. However, despite being one of the better characterized AGPCRs, the functional consequence(s) of ADGRG1 in the heart has yet to be defined. Notably, prior literature identified ADGRG1 as a mediator of mechanical overload-induced skeletal muscle hypertrophy *in vivo* and skeletal myoblast fusion *in vitro*, as well as angiotensin II-mediated NRVM hypertrophy *in vitro* [18–20]. Indeed, our scRNASeq analysis confirmed ADGRG1 expression in adult murine CM at a level and frequency similar to the well-studied angiotensin II type 1a receptor (AT1AR), and like AT1AR, its expression decreased over time in response to pressure overload-induced HF development. Altogether, these observations suggested that CM-expressed ADGRG1 may be capable of regulating an adaptive response to chronic stress *in vivo*, prior to their progression to HF. Thus, as an initial proof-of-concept study to determine whether AGPCRs may offer new targets for HF therapeutics, we generated a constitutive, CM-specific ADGRG1 knockout mouse by which to define the impact of ADGRG1 on the response to chronic cardiac stress.

Mice with global deletion of ADGRG1 did not display abnormalities in skeletal muscle development or a baseline phenotype in adults [18,19], suggesting that ADGRG1 does not impact embryonic or early post-natal muscle development. Indeed, our constitutive CM-ADGRG1-KO mice did not display differences in cardiac dimensions at 8 weeks of age and did not exhibit significant gravimetric (heart weight:tibia length) or immunohistologic changes (CM area, LV fibrosis), as shown in sham-operated controls. Although ADGRG1 deletion in cardiac myocytes, as in skeletal myocytes, did not alter cardiac muscle development or induce pathological remodeling at baseline, the increases in cardiac volumes and internal diameters by 10 weeks of age does suggest that basal mechanosensing of ADGRG1 may play a role in maintaining CM functional homeostasis, the mechanism(s) of which are unclear. As a GPCR that has primarily been shown to couple to and signal through G_12/13_, including in skeletal muscle myotubes [10,14,18,34], dynamic strain associated with normal cardiac function could regulate contractility via regulation of myofilament protein phosphorylation [37]. While activation of G_12/13_ proteins does not induce second messenger generation, it engages the small GTPase RhoA as its effector, activation of which has been shown by us to engage downstream regulators of myofilament phosphorylation, thereby promoting CM contractility in a Ca^2+^-independent manner [23,38]. The potential role of an ADGRG1-G_12/13_ protein-RhoA-dependent signaling axis that can regulate basal contractile dynamics in CM remains to be tested.

Although the changes in LV volumes and internal diameters in CM-ADGRG1-KO mice by 10 weeks of age were small compared to those normally observed in response to chronic cardiac stress (for instance in C57BL6/J mice in response to TAC [Supplementary Figure S1D–G)), they may reflect the onset of a basal stress in the absence of ADGRG1 that increases their susceptibility to chronic stress-induced dysfunction following TAC. However, the more likely explanation for the accelerated decline in function post-TAC in CM-ADGRG1-KO mice is a lack of adaptive hypertrophy since ADGRG1 was previously shown to regulate mechanical overload-induced skeletal muscle hypertrophy *in vivo* and angiotensin II-mediated NRVM hypertrophy *in vitro* [18,20]. Mechanistically, type III collagen-mediated stimulation of ADGRG1 in primary skeletal myotubes led to a G_12/13_ protein-dependent increase in growth factor expression, including IGF-1, with downstream activation of mTOR and S6K signaling that promoted an increase in myotube diameter [18]. Notably, the ADGRG1 ligand transglutaminase 2 was subsequently shown to mediate myotube hypertrophy but did not do so via IGF-1 up-regulation, instead it directly activated Akt-mTOR-p70S6K signaling [39]. While angiotensin II-mediated NRVM hypertrophy was shown to rely on ADGRG1, the downstream mechanistic underpinnings were not reported [20]. However, it seems likely in our study that in the early weeks post-TAC, the CM-ADGRG1-KO mice cannot engage an adaptive hypertrophy signaling response like those reported in skeletal myotubes, thereby preventing the heart from adjusting to the increased chronic stress. CM, which are the largest contributor to heart volume and mass, are known to increase in size as an anabolic response to pressure or mechanical overload [30,31,40] and indeed, the αMHC-Cre control mice displayed increased LV wall thicknesses at 3 weeks post-TAC, during the compensatory hypertrophy phase, while the CM-ADGRG1-KO mice lacked this response.

Whether via lack of an adaptive hypertrophic response to chronic stress or another mechanism, CM-specific loss of ADGRG1 ultimately accelerated HF progression, specifically toward a dilated phenotype. At the end of the TAC experiments, the hearts were removed and their wet weights attained for normalization to tibia length. Since the CM-ADGRG1-KO TAC hearts had higher HW/TL values without increased LV thickness or cardiomyocyte hypertrophy, this likely reflects increased fluid retention, which would be expected of sicker cardiomegalic hearts unable to pump effectively [41], as corroborated by the higher systolic LV volume and diameter by this timepoint. In addition to LV dilation, there was significant accumulation of peripheral immune cells by 5-week post-TAC, indicating an increased cardiac inflammatory response in conjunction with the development of dysfunction. While the specific immune cell populations recruited to the heart in this model await follow-up study, an accumulation of inflammatory monocytes, monocyte-derived macrophages and T cells would be expected [42]. Notably, although cardiac inflammation promotes fibrosis, at 5-week post-TAC we did not observe a difference in LV fibrosis via either MTC or αSMA staining between the CM-ADGRG1-KO and αMHC-Cre groups, though we expect that if the CM-ADGRG1-KO mice could survive longer, they would likely develop more fibrosis in response to the heightened peripheral immune cell infiltration. Thus, CM-specific loss of ADGRG1 is associated with accelerated contractile dysfunction and lack of adaptive remodeling under conditions of chronic stress, thereby leading to severe HF and enhanced mortality.

Despite showing the relatively large phenotypic effect of CM-specific ADGRG1 deletion on TAC-induced HF, a limitation of our study relates to understanding how it mediates this response regionally. Spatiotemporal profiling of CM-specific ADGRG1 expression within the heart normally and during the development and progression of HF would be informative since our scRNASeq analysis revealed that ∼20% of CMs express ADGRG1 in healthy hearts, which is reduced 10-fold, down to ∼2% of CMs in mice that progressed to HF in the pressure overload model. This profile suggests that there may exist a subpopulation of CMs that responds to mechanical stress associated with the normal function of the heart in a particular region of the heart that can detect and respond quickly to changes in LV pressure overload. Understanding where CM are located that express ADGRG1 and what becomes of them once the heart progresses to failure would be informative. Importantly, ADGRG1 has been shown to possess a variety of modes of receptor activation, including via binding to its known ligands type III collagen, transglutaminase 2 and tetraspanins, as well as mechanosensitive activation in response to mechanical stress [36,43]. Our data showed that expression of both type III collagen and transglutaminase 2 increase at 6 weeks post-TAC in wild-type mice, the source of which we expect to be cardiac fibroblasts [16,17,44,45]. However, whether this increased expression occurs specifically in activated fibroblasts, or another cell type present at that timepoint, and in what location relative to the CM expressing ADGRG1, would help define the inter-cellular ligand-receptor dynamic that orchestrates the impact of CM-specific ADGRG1 signaling in the heart.

## Supplementary Material

Supplementary Figures S1-S4 and Table S1

## Data Availability

All study data are available from the corresponding author upon request.

## Abbreviations

ADGRG1: adhesion G protein-coupled receptor G1
AGPCR: adhesion G protein-coupled receptor
CM: cardiomyocyte
Col3a1: type III collagen
HF: heart failure
LV: left ventricular
MHC: myosin heavy chain
Tg2: transglutaminase
