# Supplementary material for: Loss of cardiomyocyte-specific adhesion G-protein-coupled receptor G1 (ADGRG1/GPR56) promotes pressure overload-induced heart failure
Source: Biosci Rep. 2024 Sep 23;44(9):BSR20240826. doi: 10.1042/BSR20240826 (PMC11427730; doi:10.1042/BSR20240826)
Supplement: Supplementary Figures S1-S4 and Table S1 [file BSR-2024-0826_supp.pdf]

Supplementary Information

Supplemental Table 1. RTqPCR Primers			
**Gene**	**Species**	**Forward**	**Reverse**
Adgrg1	Mouse	5’ – TTGCTGACCTCTGCTCC –3’	5’ – AGCAGGAAGACAGCGGACAG –3’
Tg2	Mouse	5’–CGAATCCTCTACGAGAAGTACAGC–3’	5’–CAGTTTGCGGTTTTGCTTGG–3’
Col3a1	Mouse	5' -CCTGGTGGAAAGGGTGAAAT - 3'	5' -CGTGTTCCGGGTATACCATTAG - 3'
Tpt1	Mouse	5' -ATCATCTACCGGGACCTCATC - 3'	5' -CCCTCTGTTCTACTGACCATCT - 3'

Supplemental Figure Legends

Supplemental Figure 1. Echocardiographic measurements of wild-type mice following TAC. Serial echocardiography was performed at baseline, 6- and 12-weeks post-surgery to assess heart rate (HR, A), stroke volume (SV, B), cardiac output (CO, C), systolic LV volume (LV Vol s, D), diastolic LV volume (LV Vol d, E), systolic LV internal diameter (LVID s, F), diastolic LV internal diameter (LVID d, G), systolic LV posterior wall thickness (LVPW s, H), diastolic LV posterior wall thickness (LVPW d, I), systolic LV anterior wall thickness (LVAW s, J), diastolic LV anterior wall thickness (LVAW d, K) and LV mass (L). Data are mean ± SEM, *P < 0.05, **P < 0.01, ***P < 0.001, two-way ANOVA with Tukey's post-hoc test, n=8 male (baseline, 6 weeks) or 4 male (12 weeks) mice for Sham and TAC groups.

Supplemental Figure 2. Echocardiographic and gravimetric measurements of CM-ADGRG1-KO and αMHC-Cre mice. Serial echocardiography was performed at 8 and 10 weeks of age to assess heart rate (HR, A), systolic LV posterior wall thickness (LVPW s, B), diastolic LV posterior wall thickness (LVPW d, C), systolic LV anterior wall thickness (LVAW s, D), diastolic LV anterior wall thickness (LVAW d, E) and LV mass (F). Data are mean ± SEM, two-way ANOVA with Tukey's post-hoc test, n=5 per group. (G) Heart weight/tibia length ratios were assessed at 12 weeks of age in CM-ADGRG1-KO and αMHC-Cre mice. Data are mean ± SEM, ns = not significant, two-tailed t-test, n=5; 4 female, 1 male (CM-ADGRG1-KO) and n=5; 3 female, 2 male (αMHC-Cre).

Supplemental Figure 3. Echocardiographic measurements of CM-ADGRG1-KO and αMHC-Cre mice following TAC. Serial echocardiography was performed at baseline, 1-, 3- and 5-weeks post-surgery to assess heart rate (HR, A), stroke volume (SV, B), cardiac output (CO, C), systolic LV volume (LV Vol s, D), diastolic LV volume (LV Vol d, E), systolic LV internal diameter (LVID s, F), diastolic LV internal diameter (LVID d, G), systolic LV posterior wall thickness (LVPW s, H), diastolic LV posterior wall thickness (LVPW d, I), systolic LV anterior wall thickness (LVAW s, J), diastolic LV anterior wall thickness (LVAW d, K) and LV mass (L). Data are mean ± SEM, *P < 0.05, **P < 0.01, ***P < 0.001 CM-ADGRG1-KO TAC versus MHC-Cre TAC, #P<0.05, ##P < 0.01, ###P < 0.001 CM-ADGRG1-KO Sham versus CM-ADGRG1-KO TAC, †P<0.05, ††P < 0.01 MHC Cre+ Sham versus MHC Cre+ TAC and ^P<0.05, ^^P<0.01, ^^^P<0.001 MHC Cre+ Sham versus CM-ADGRG1-KO Sham, two-way ANOVA with Tukey's post-hoc test, n=4 (MHC-Cre Sham; 2 male, 2 female), n=5 (CM-ADGRG1-KO Sham; 1 male, 4 female), n=6 (MHC-Cre TAC; 3 male, 3 female), n=7 (CM-ADGRG1-KO TAC; 2 male, 5 female).

Supplemental Figure 4. Cardiomyocyte-specific ADGRG1 deletion does not impact αSMA accumulation in response to chronic pressure overload. αSMA staining was performed on cardiac slices from CM-ADGRG1-KO and MHC Cre mice at 5 weeks post-surgery, with data quantified in histogram. Data are mean ± SEM, ns = not significant, one-way ANOVA with Tukey's post-hoc test. n=4 (MHC-Cre Sham; 2 male, 2 female), n=5 (CM-ADGRG1-KO Sham; 1 male, 4 female), n=6 (MHC-Cre TAC; 3 male, 3 female), n=6 (CM-ADGRG1-KO TAC; 2 male, 4 female).

Supplemental Figure 1

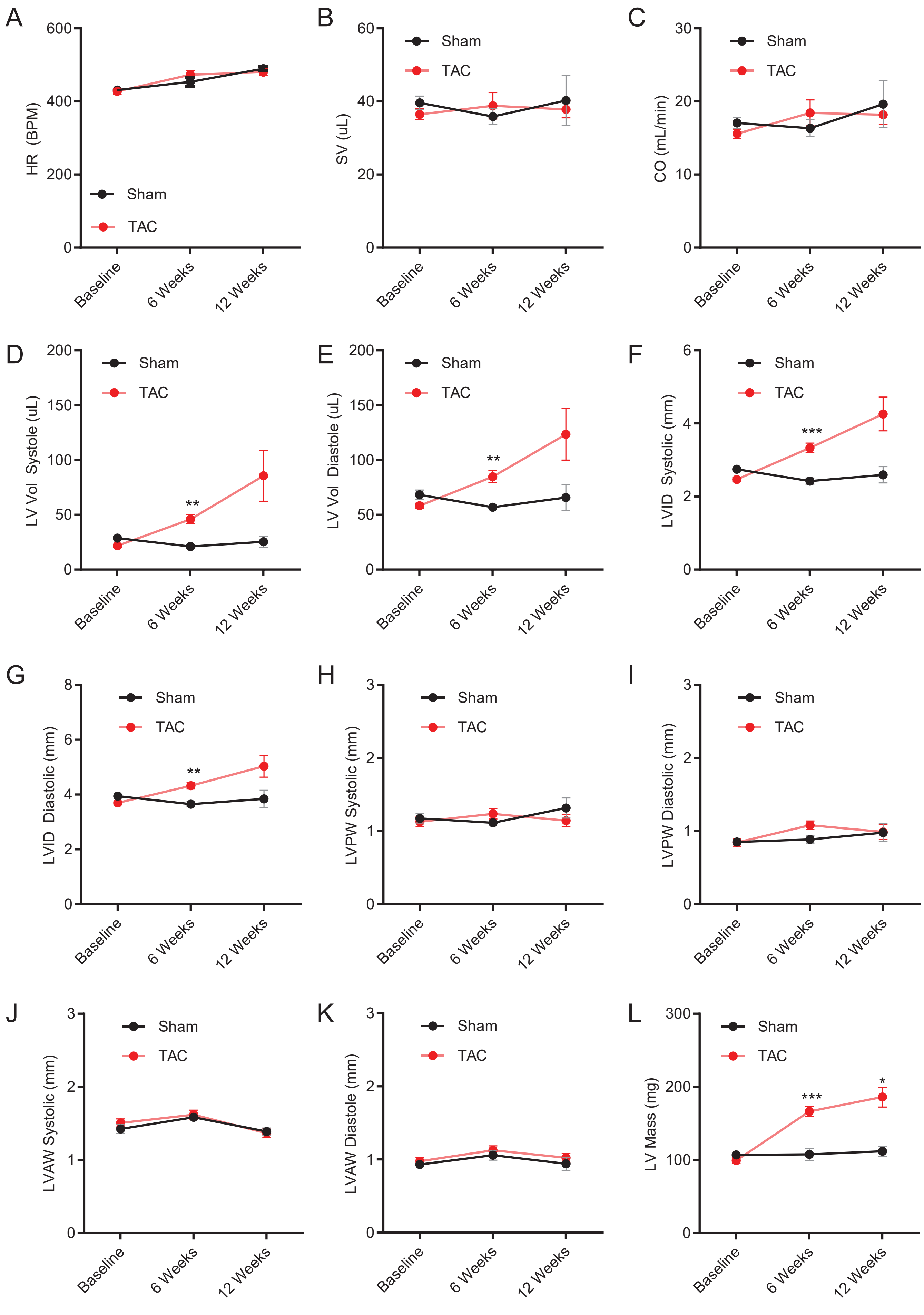

Supplemental Figure 2

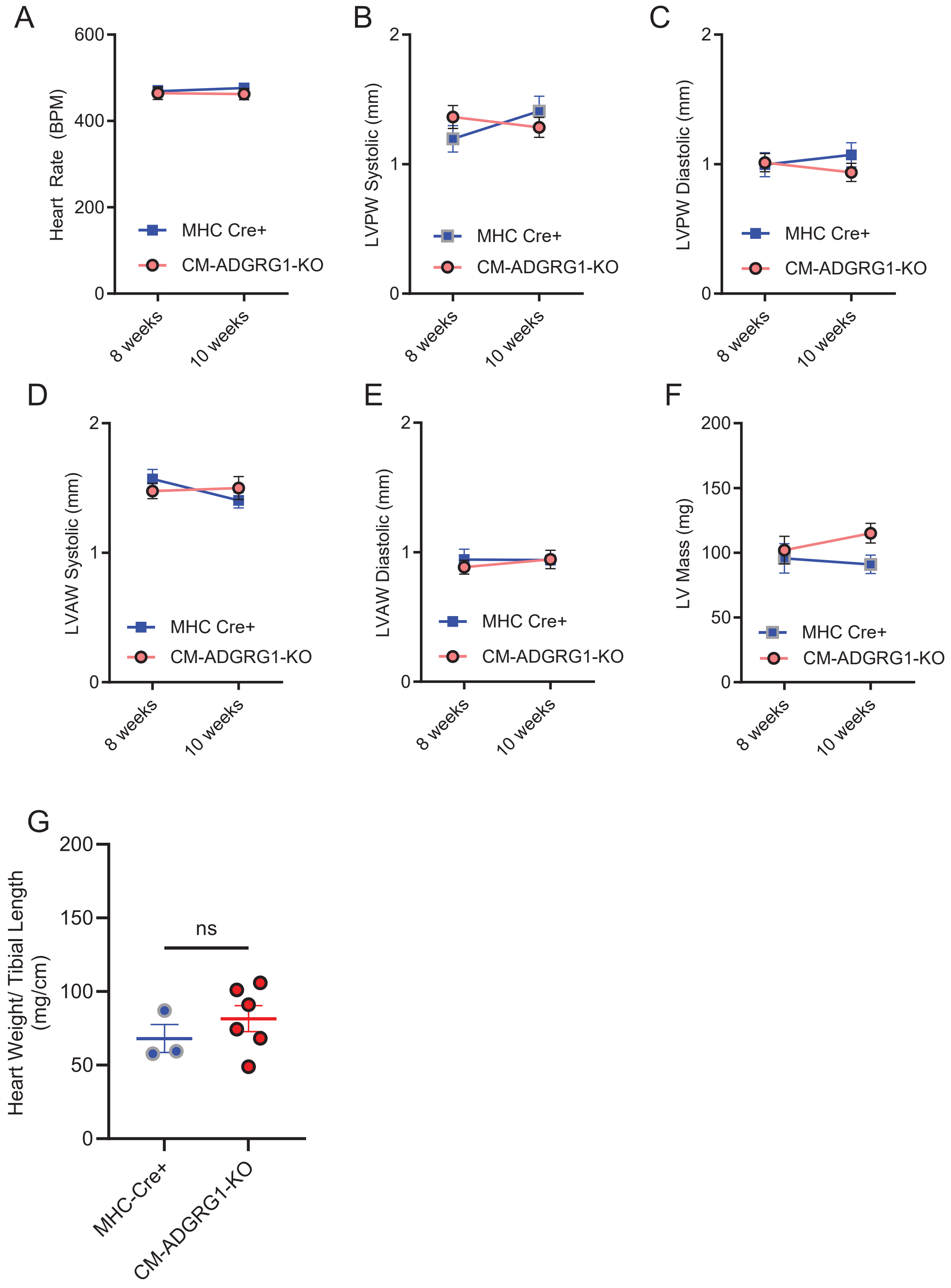

Supplemental Figure 3

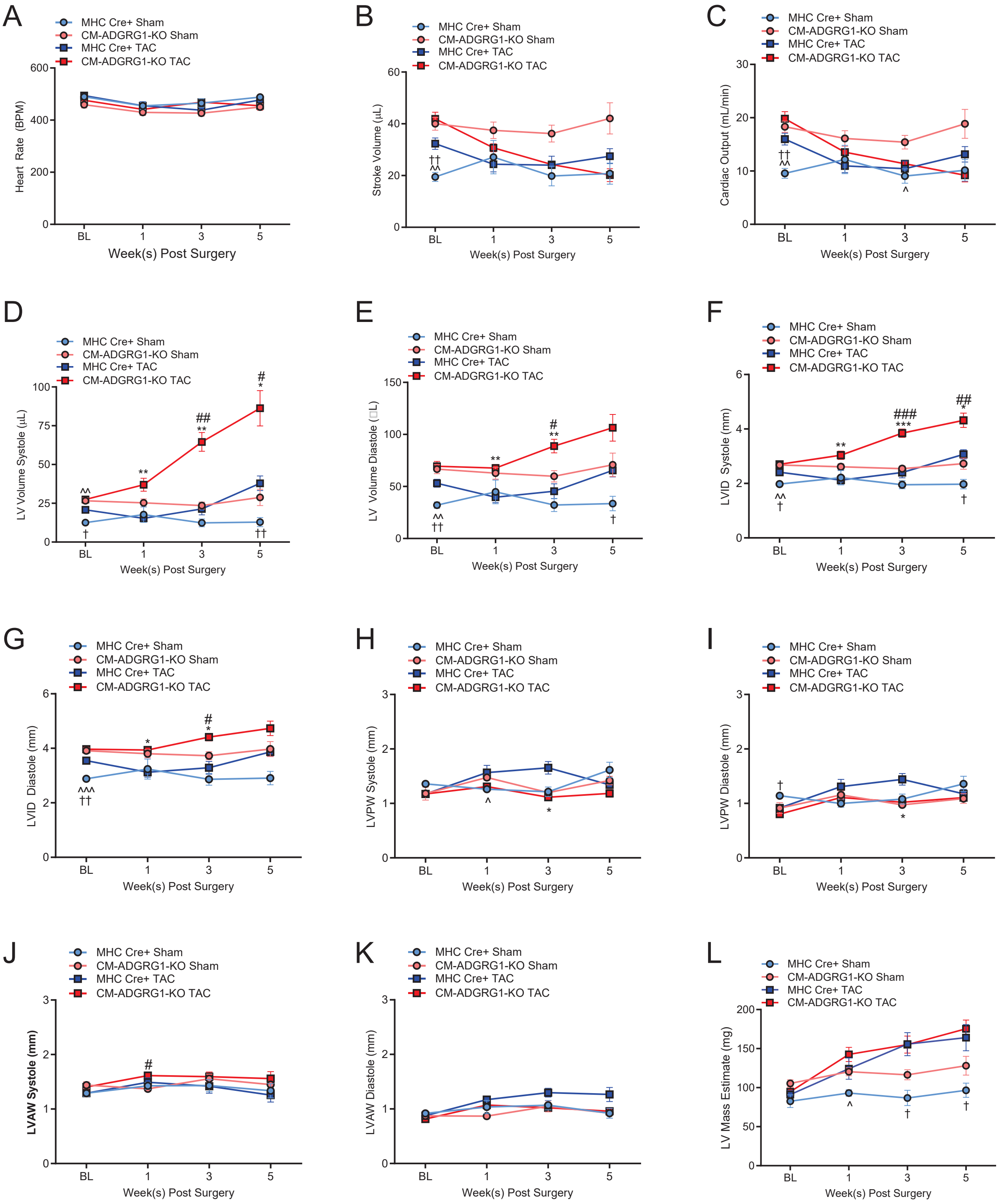

Supplemental Figure 4

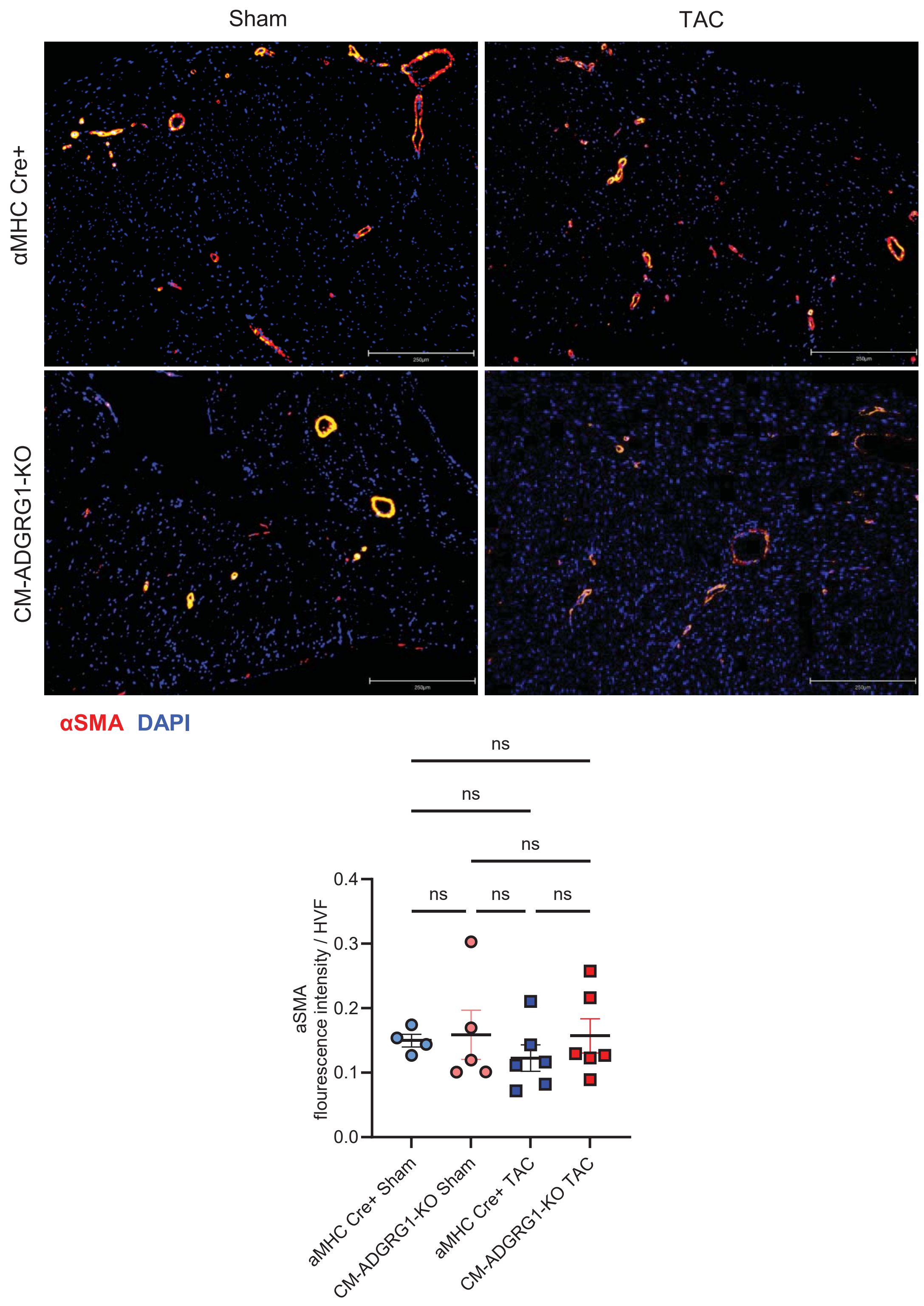

Full Uncropped Blots for validation of ADGRG1 expression in primary adult mouse cardiomyocytes

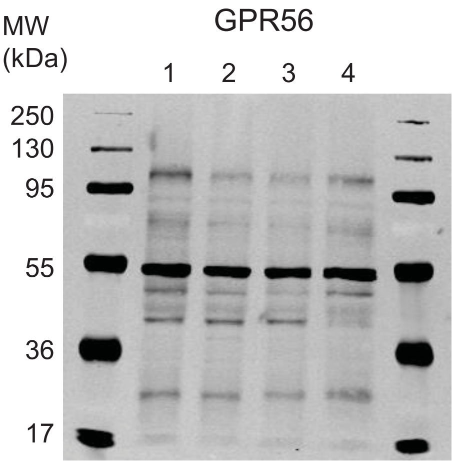

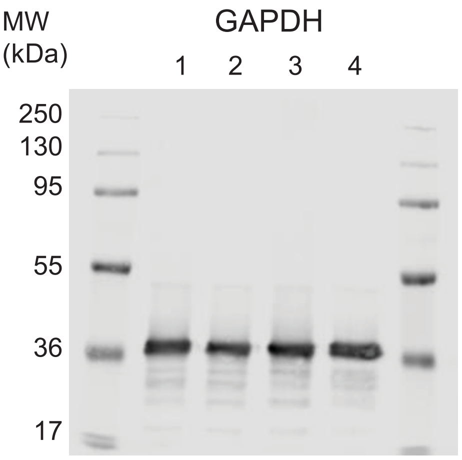

Samples:
1. 6149 - ADGRG1$^{f/f}$
2. 6151 - ADGRG1$^{f/f}$
3. 6150 - CM-ADGRG1-KO
4. 6152 - CM-ADGRG1-KO

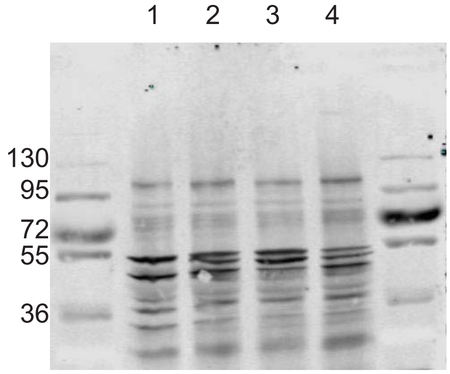

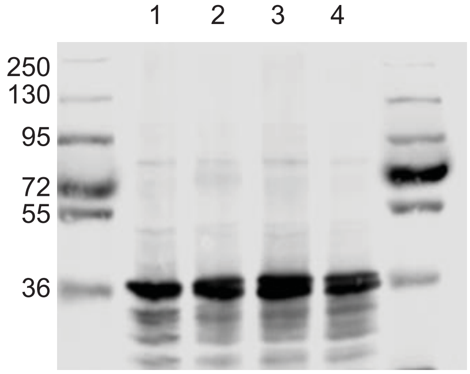

Samples:
1. 6594 - ADGRG1$^{f/f}$
2. 6595 - ADGRG1$^{f/f}$
3. 6592 - CM-ADGRG1-KO
4. 6597 - CM-ADGRG1-KO

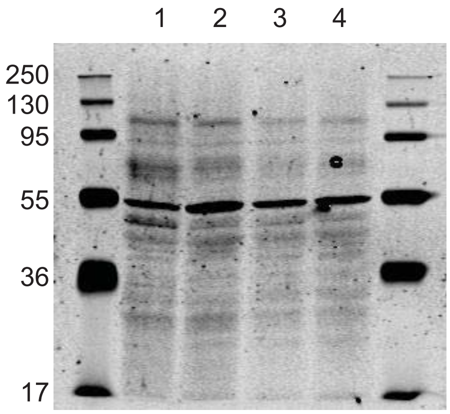

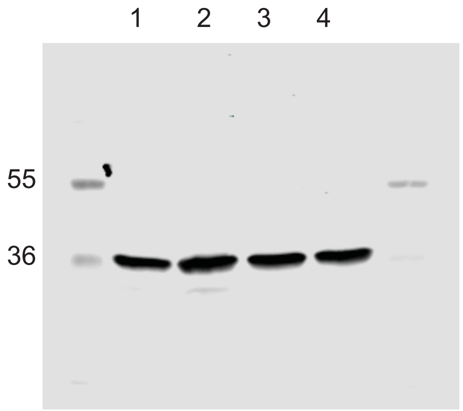

Samples:
1. 6654 - ADGRG1$^{f/f}$
2. 6691 - ADGRG1$^{f/f}$
3. 6653 - CM-ADGRG1-KO
4. 6690 - CM-ADGRG1-KO

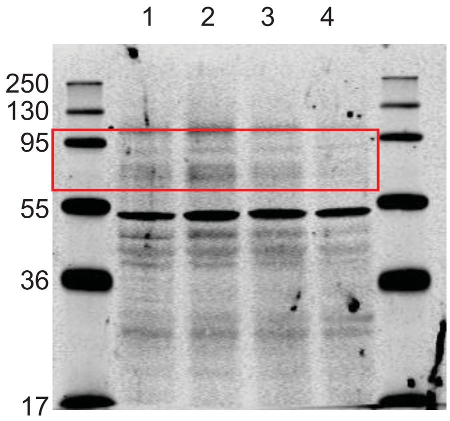

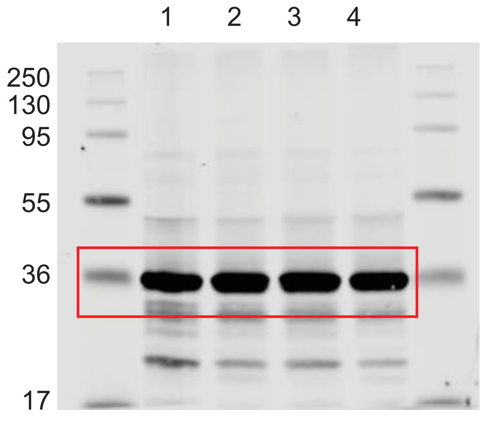

Samples:
1. 6689 - ADGRG1$^{f/f}$
2. 6692 - ADGRG1$^{f/f}$
3. 6688 - CM-ADGRG1-KO
4. 6693 - CM-ADGRG1-KO

**These blots used for Figure 1*
